# Factors influencing the overall satisfaction of teachers participating in a nationwide school-based smoking prevention program in Korea

**DOI:** 10.18332/tid/190067

**Published:** 2024-07-10

**Authors:** Geon Heo, Seulgi Kim, Sung-il Cho, Seunghyun Yoo, Jieun Hwang

**Affiliations:** 1 Department of Health Administration, Dankook University, Cheonan, Republic of Korea; 2 Institute of Health and Environment, Seoul National University, Seoul, Republic of Korea; 3 Department of Public Health Sciences, Graduate School of Public Health, Seoul National University, Seoul, Republic of Korea

**Keywords:** school, smoking, prevention program, teacher, satisfaction

## Abstract

**INTRODUCTION:**

Numerous studies of school-based smoking prevention programs (SSPPs) exist; however, most have been conducted from the students' perspective, and insufficient research has explored teachers' perceptions. Our study aimed to identify factors affecting overall satisfaction and operational status from the perspective of teachers participating in the SSPP.

**METHODS:**

This is a cross-sectional study analyzing data from a survey regarding the operation of an SSPP conducted by the Korea Health Promotion Institute in 2022. The study sample comprised 669 teachers involved in the SSPP: 215 from elementary schools, 212 from middle schools, and 242 from high schools. To identify factors influencing teachers’ overall satisfaction, independent variables were categorized into three types of factors: personal, school, and teacher perceptions. Multiple linear regression analysis was performed for each factor to test the independent association.

**RESULTS:**

For elementary school teachers, as the necessity of smoking prevention and cessation education (β=0.292; 95% CI: 0.182–0.382) increased, the overall satisfaction with the operation of the SSPP significantly improved. Similarly, for middle school teachers, as the necessity of smoking prevention and cessation education (β=0.231; 95% CI: 0.104–0.336) increased, the overall satisfaction significantly improved. Conversely, for high school teachers, the effectiveness (β=0.347; 95% CI: 0.184–0.520) was the variable that significantly improved overall satisfaction with the SSPP operation. The variables affecting teacher satisfaction across all school levels were the necessity of smoking prevention and cessation education, the effectiveness of the SSPP, and its impact on smoking cessation among smoking students, all of which belonged to the teacher perceptions factor.

**CONCLUSIONS:**

Smoking education in schools requires teachers to play a crucial role. Among participating teachers, overall satisfaction with SSPP operations and the influencing factors differed according to school level, highlighting the importance of careful consideration to establish a more effective operational environment tailored to each school level.

## INTRODUCTION

Smoking is recognized for its harmful effects on the human body, particularly as a risk factor for severe conditions such as cardiovascular disease (CVD), and chronic obstructive pulmonary disease^1^. Smoking during childhood and adolescence poses a greater risk in terms of addiction, CVD severity, and airflow obstruction compared with smoking in adulthood^2^. Considering the association of smoking during adolescence with a higher likelihood of becoming a daily smoker in adulthood^3^, various policies have been implemented to prevent smoking initiation among adolescents. Despite a decrease in the prevalence of smoking among adolescents^4^, new forms of tobacco, such as e-cigarettes, hookah, and smokeless tobacco, have emerged; their use rates are steadily increasing^4-6^. Management of these alternatives has become a key focus of adolescent smoking prevention education policies.

As the prevalence of new forms of tobacco use among adolescents continues to rise, many countries worldwide have implemented school-based smoking prevention programs (SSPPs)^7,8^.

In Greece, a student participatory SSPP was implemented targeting grades 1–3 of middle school through role-playing based on Greek mythology and debates on smoking policies^7^. In the study, students were divided into intervention and control groups based on whether they received the SSPP; a survey was conducted before and after a 3-month period^7^. The results showed that although there were no significant changes in smoking indicators in the control group, the intervention group experienced increased awareness of the harmful effects of smoking and a significant reduction in future smoking intentions^7^.

In Malaysia, an SSPP was conducted targeting non-smoking fifth-grade students, segmented into sessions focusing on base knowledge, social influence, and perceived behavioral control^8^. Surveys conducted before and after SSPP implementation indicated improvement in the average scores of subjective norms regarding smoking and non-smoking intentions in the intervention group, confirming the effectiveness of the SSPP^8^.

After its inception in 1999 under private initiatives, Korea’s SSPP underwent minor changes until 2014^9^. However, a significant increase in cigarette prices led to considerable expansion of the program’s scope; it has been implemented in all elementary, middle, and high schools nationwide since 2015^9^. The SSPP aims to create a tobacco-free environment for the next generation by establishing school regulations against smoking, making declarations for smoke-free schools, providing smoking prevention education for students, and offering smoking cessation counseling hotlines^9,10^. The SSPP has successfully established a tobacco-free school environment, positively affecting intentions to quit smoking among participating students and their families^10^. Furthermore, the SSPP has contributed to steady declines in smoking initiation from adolescence and the smoking rate among Korean adolescents since 2015^9^.

Schools, led by teachers, aim to provide multifaceted education that nurtures adolescents’ cognitive functions, self-identity, and to foster successful societal integration, considering that they are the institutions where adolescents spend most of their time^11^. Because education is directly imparted to students through teachers, the professional competence of teachers significantly influences the effectiveness of students’ education^12^. Teacher job satisfaction, professional competence, and efficacy are key factors for effective student education^13,14^. Similarly, in the school health education environment, the efficacy and satisfaction of school nurse teachers positively impact health education outcomes, playing a vital role in establishing an effective school health education environment^15,16^.

Although studies have explored the satisfaction and effectiveness of school nurse teachers’ education^15,17^, research from the perspective of teachers participating in the SSPP is limited to studies of teaching modules^18,19^. Previous SSPP research has predominantly focused on intervention effectiveness from students’ perspectives^7,8^, overlooking the direct assessment of leading teachers’ overall satisfaction, workload, and educational environment. Teachers are at the forefront of SSPP education; thus, the overall satisfaction of participating teachers in these programs is expected to closely associated with achievement of the program’s objectives^10,15,16^.

Our study examined the operational status of Korea’s SSPP across various school levels based on survey results to identify the satisfaction levels of participating teachers according to school level and to determine the factors influencing program operation satisfaction. Our findings offer insights for future SSPP initiatives tailored to each school level.

## METHODS

### Study populations and procedure

This cross-sectional study examined the current status of schools participating in the SSPP and identified factors influencing teachers’ satisfaction with its operation^10^. We utilized data from the 2022 survey regarding the operation of the SSPP conducted by the Korea HEalth Promotion Institute (KHEPI).

The survey of SSPP operations, conducted annually since 2015 by the National Tobacco Control Support Center of the KHEPI, aims to assess the operational status and identify issues useful for formulating effective program strategies. The operational survey is a self-administered online questionnaire conducted annually through a targeted sampling method that focuses on teachers and students participating in the SSPP. A total of 810 schools were randomly allocated, with 270 schools per school level. The sampling followed the principle of probability proportional to size (PPS) to ensure that the sample composition matched the population composition across stratification variables. The sampling method employed was a two-stage stratified cluster sampling. The first sampling unit was the school, and the second sampling unit was the class. One class per grade level was selected from each sampled school. Through sample design, selected teachers and students from elementary, middle, and high schools (grades 5–12) are surveyed regarding various elements including basic characteristics of the program, operational statuses of smoking prevention and cessation programs, students’ smoking-related behaviors and attitudes, teachers’ perceived effectiveness, and program satisfaction. A total of 669 teachers (215 from elementary schools, 212 from middle schools, and 242 from high schools) who participated in the operational survey were selected from a sample of 575 schools, chosen from among the 10797 schools participating in the 2022 SSPP. There were no missing data in this survey. We used anonymized public data. The study protocol was approved by the Institutional Review Board of Seoul University (IRB No. E2311/004-003). All participants provided written informed consent.

### Overall satisfaction with operation of the SSPP

Teachers were asked: ‘How satisfied are you overall with the operation of the SSPP?’ with response options on a 5-point Likert scale (1=‘very dissatisfied’, 2=‘dissatisfied’, 3=‘neutral’, 4=‘satisfied’, and 5=‘very satisfied’).

### Personal factors

Sex, age, and experience in the SSPP were regarded as personal factors that could influence satisfaction. Categories for sex and age were: sex (male, female) and age (divided into 20s, 30s, 40s, and 50s and above). Experience in the SSPP was assessed by asking: ‘How long have you been in charge of the SSPP?’ with response options: <1, ≥1 and <2, ≥2 and <3, ≥3 and <4, and ≥4 years.

### School factors

Independent variables related to the characteristics of schools participating in the SSPP were regarded as school factors. Programs were categorized as: ‘Basic type’ and ‘Advanced type’. The schools participating in the SSPP were divided into basic and advanced types. The advanced type receives a higher budget allocation compared to the basic type and implements a greater number of school-based smoking prevention programs. The locations of schools were divided into three categories: capital region (Seoul, Gyeonggi, or Incheon), metropolitan region (Daejeon, Daegu, Gwangju, Ulsan, or Busan, excluding Incheon), and other local government regions. School size, based on the number of enrolled students, was measured in increments of 100 students, ranging from fewer than 100 to ≥1000. We categorized <400 students as small schools, ≥400 and <1000 students as medium-sized schools, and ≥1000 students as large schools. The budget was analyzed based on the categories: <50, ≥50 and <250, ≥250 and <500, ≥500 and <750, ≥750 and <1000, and ≥1000 considering all monetary values in 10 thousand KRW (1000 Korean Won about US$0.73).

The levels of interest and support from school administrators (principal) and school officials (board members) regarding school smoking prevention activities, as well as the interest levels of regular teachers, were measured on a 5-point Likert scale (1=’almost none’, 2=‘not much’, 3=‘average’, 4=‘somewhat significant’, and 5=‘very significant’). The number of teachers actively participating in school smoking prevention activities was categorized as: alone, 2–3, 4–5, and ≥6 teachers.

### Teacher perceptions factors

Factors perceived by teachers, which could influence their satisfaction with the operation of the SSPP, were categorized as perceptions factors. Even teachers who reported no smoking-related policies were presumed to have some policies in place. They were then asked: ‘Do you think smoking-related school rules are helpful for smoking prevention and cessation?’ to assess the extent to which smoking rules contribute to cessation efforts. Responses were assessed by: ‘Not helpful at all’, ‘Not very helpful’, ‘Neutral’, ‘Somewhat helpful’, and ‘Very helpful’. The burden of preparing educational materials was categorized as: ‘Very burdensome’, ‘Slightly burdensome’, ‘Neutral’, ‘Not very burdensome’, and ‘Not burdensome at all’. The necessity of smoking prevention and cessation education was categorized as: ‘Not needed at all’, ‘Not really needed’, ‘Neutral’, ‘Somewhat needed’, and ‘Very much needed’. The effectiveness of the SSPP, its smoking prevention effect on non-smoking students, and its smoking cessation effect on smoking students were categorized as: ‘Almost no effect’, ‘Not really effective’, ‘Neutral’, ‘Somewhat effective’, and ‘Very effective’.

### Statistical analysis

To understand the demographic characteristics of participating teachers and the characteristics of the schools in which they worked, frequency analysis and cross-tabulations were conducted. Differences at the school level were analyzed using the Rao-Scott χ^2^ test. We conducted the Shapiro-Wilk test to determine whether the data deviated from a normal distribution. Additionally, to check for the independence of residuals, we performed the Durbin-Watson test. We performed one-way analysis of variance (ANOVA) with Bonferroni *post hoc* tests to compare teachers’ overall satisfaction with SSPP operations across school levels. Pearson correlation analysis was utilized to examine correlations among variables. Multiple linear regression analysis was conducted to check for multicollinearity among independent variables and to identify factors influencing teachers’ satisfaction with SSPP operations based on school level. After adjusting for personal factors and school factors, standardized coefficients (β coefficients) and 95% confidence intervals (CIs) are presented. All statistical analyses were performed using the survey modules of SAS statistical software version 9.4 (SAS Institute, Inc., Cary, NC, USA). Values of p<0.05 were considered statistically significant.

## RESULTS

### General characteristics of teachers participating in the SSPP

The total number of teachers participating in the SSPP Operation Survey was 669, comprising 215 (32.1%) elementary school teachers, 212 (31.7%) middle school teachers, and 242 (36.2%) high school teachers (Table 1). Among these participants, teachers aged ≥50 years constituted the largest proportion (35.9%) in the age groups. As age increased, the proportion also gradually increased; elementary school teachers constituted the largest percentage at 37.5% among those in their 50s, whereas high school teachers comprised the largest proportion among those in their 20s to 40s (p=0.007).

**Table 1:** Characteristics of teachers participating in the SSPP operation survey, a cross-sectional study, Korea, 2022 (N=699)

*Characteristics*	*Total (N=669)*		*School level*						p*
			*Elementary school (N=215)*		*Middle school (N=212)*		*High school (N=242)*		
	*n*	*%*	*n*	*%*	*n*	*%*	*n*	*%*	
**Total**	669	100	215	32.1	212	31.7	242	36.2	
**Sex**									<0.0001
Male	128	19.1	8	6.2	44	34.4	76	59.4	
Female	541	80.9	207	38.3	168	31.0	166	30.7	
**Age** (years)									0.007
20–29	53	7.9	17	32.1	13	24.5	23	43.4	
30–39	140	20.9	37	26.4	46	32.9	57	40.7	
40–49	236	35.3	71	30.1	66	28.0	99	41.9	
≥50	240	35.9	90	37.5	87	36.2	63	26.2	
**School location**									0.624
Capital region	207	30.9	71	34.3	64	30.9	72	34.8	
Metropolitan city	136	20.3	36	26.5	47	34.6	53	39.0	
Local government	326	48.7	108	33.1	101	31.0	117	35.9	
**Size of school**									<0.0001
<400 (small)	319	47.7	118	37.0	119	37.3	82	25.7	
400–999 (medium-sized)	278	41.5	70	25.2	81	29.1	127	45.7	
≥1000 (large)	72	10.8	27	37.5	12	16.7	33	45.8	
**Type of SSPP**									0.045
Basic	584	87.3	192	32.9	191	32.7	201	34.4	
Advanced	85	12.7	23	27.1	21	24.7	41	48.2	
**Program participation** (years)			**1**						<0.0001
<1	176	26.3	30	17.0	53	30.1	93	52.8	
1≤ and <2	92	13.7	25	27.2	31	33.7	36	39.1	
2≤ and <3	64	9.6	15	23.4	19	29.7	30	46.9	
3≤ and <4	44	6.6	10	22.7	18	40.9	16	36.4	
≥4	293	43.8	135	46.1	91	31.1	67	22.9	

* Rao-Scott χ^2^. SSPP: school-based smoking prevention program.

### Differences in overall satisfaction with the SSPP according to school level

We performed one-way ANOVA to statistically compare teachers’ overall satisfaction with the operation of the SSPP across school levels (Table 2). The degree of assistance from smoking-related school policies was lowest in elementary schools, with an average score of 3.45, and highest in high schools, with a score of 3.98. The burden of preparing educational materials for the SSPP was reflected by an overall average score of 2.31, indicating that teachers generally felt burdened by this task. The effectiveness of the SSPP, its smoking prevention effect on non-smoking students, and its smoking cessation effect on smoking students showed the highest ratings for elementary schools and decreased with increasing school grade level (p<0.001). Overall satisfaction with the operation of the SSPP among participating teachers was highest for high schools at 3.61 points and lowest for middle schools at 3.44 points.

**Table 2:** Means and standard deviations of 5-point Likert variables, a cross-sectional study, Korea, 2022 (N=699)

*Characteristics*	*Total (N=669)*		*School level*						p*	*Bonferroni*
			*Elementary school ^a^ (N=215)*		*Middle school ^b^ (N=212)*		*High school ^c^ (N=242)*			
	*Mean*	*SD*	*Mean*	*SD*	*Mean*	*SD*	*Mean*	*SD*		
School interest in the SSPP	3.64	1.072	3.56	1.108	3.71	0.988	3.65	1.110	0.374	
General teachers’ interest in the SSPP	3.49	1.033	3.42	1.046	3.50	0.990	3.54	1.059	0.486	
School smoking ban policy assistance	3.76	0.958	3.45	1.030	3.83	0.883	3.98	0.888	<0.0001	a < b, c
Burden of preparing educational materials for the SSPP	2.31	0.920	2.42	0.875	2.44	0.906	2.08	0.973	0.084	
Necessity of smoking prevention and cessation education	4.10	0.838	4.13	0.872	4.06	0.844	4.11	0.802	0.658	
Effectiveness of the SSPP	3.63	0.792	3.78	0.725	3.58	0.772	3.54	0.850	<0.0001	a > b, c
Smoking prevention effect on non-smoking students	3.87	0.787	4.05	0.775	3.86	0.740	3.72	0.806	<0.0001	a > b, c
Smoking cessation effect on smoking students	3.52	0.967	3.68	1.028	3.47	0.910	3.43	0.945	<0.0001	a > b, c
Overall satisfaction	3.54	0.840	3.58	0.844	3.44	0.844	3.61	0.844	0.0005	a, b < c

* One-way analysis of variance. SSPP: school-based smoking prevention program.

Bonferroni correction: a) elementary school; b) middle school; c) high school.

### Empirical analysis of factors influencing the satisfaction of participating teachers with the operation of the SSPP

We utilized Pearson correlation analysis to examine factors influencing satisfaction among teachers participating in the SSPP (Table 3). Correlation analysis results revealed that overall satisfaction was positively correlated with the school’s interest in the SSPP (r=0.434, p<0.001), general teachers’ interest in the SSPP (r=0.495, p<0.001), the perceived assistance of smoking policies (r=0.416, p<0.001), the burden of preparing educational materials (r=0.495, p<0.001), the necessity of smoking prevention and cessation education (r=0.495, p<0.001), the effectiveness of the SSPP (r=0.605, p<0.001), smoking prevention effect on non-smoking students (r=0.504, p<0.001), and smoking cessation effect on smoking students (r=0.529, p<0.001).

**Table 3:** Pearson correlation (r) between 5-point Likert scale variables, a cross-sectional study, Korea, 2022 (N=699)

*Variables*	*1*	*2*	*3*	*4*	*5*	*6*	*7*	*8*	*9*
1. Overall satisfaction	-								
2. School interest in the SSPP	0.434***	-							
3. General teachers’ interest in SSPP	0.495***	0.739***	-						
4. School smoking policy assistance	0.416***	0.345***	0.367***	-					
5. Burden of preparing educational materials for SSPP	0.300***	0.176***	0.213***	0.134***	-				
6. Necessity of smoking prevention and smoking education	0.495***	0.309***	0.342***	0.314***	0.204***	-			
7. Effectiveness of SSPP	0.605***	0.378***	0.485***	0.404***	0.203***	0.386***	-		
8. Smoking prevention effect on non-smoking students	0.504***	0.393***	0.428***	0.323***	0.197***	0.372***	0.676***	-	
9. Smoking cessation effect on smoking students	0.529***	0.359***	0.430***	0.353***	0.186***	0.291***	0.639***	0.563***	-

SSPP: school-based smoking prevention program.

* p<0.05,

** p<0.01,

*** p<0.001.

To identify factors influencing overall satisfaction among teachers participating in the SSPP according to school levels, we conducted multiple regression analysis with independent variables categorized into personal factors, school characteristics factors, and teacher perceptions factors (Table 4).

**Table 4:** Factors associated with the overall satisfaction of the SSPP operation, a cross-sectional study, Korea, 2022 (N=699)

*Independent variables*	*School*											
	*SSPP participating (N=669)*			*Elementary school (N=215)*			*Middle school (N=212)*			*High school (N=242)*		
	*Coef*	*SE*	*β 95% CI*	*Coef*	*SE*	*β 95% CI*	*Coef*	*SE*	*β 95% CI*	*Coef*	*SE*	*β 95% CI*
**Personal factors**												
Sex (male)	-0.037	0.062	-0.017 -0.160–0.085	0.107	0.216	0.024 -0.319–0.532	-0.002	0.028	-0.001 -0.226–0.221	-0.004	0.088	-0.002 -0.178–0.170
Age (years)	-0.006	0.267	-0.006 -0.058 – -0.046	**0.125**	**0.047**	0.141** **0.032–0.218**	-0.058	0.050	-0.068 -0.158–0.041	-0.043	0.445	-0.046 -0.132–0.046
Program participation (years)	0.009	0.062	0.017 -0.022–0.039	-0.014	0.216	-0.026 -0.075–0.047	0.021	0.028	0.041 -0.036–0.075	0.023	0.025	0.045 -0.026–0.072
**School factors**												
Location of school (capital region)	-0.003	0.004	-0.002 -0.012–0.005	0.008	0.008	0.045 -0.007–0.022	0.003	0.008	0.021 -0.013–0.019	**-0.016**	**0.007**	-0.111* **-0.134 – -0.035**
Size of school	**-0.015**	**0.008**	-0.059* **-0.081 – -0.006**	**-0.031**	**0.014**	-0.128* **-0.186 – -0.024**	-0.024	0.017	-0.091 -0.057–0.009	0.007	0.013	0.026 -0.019–0.033
Type of SSPP (basic)	0.006	0.092	0.002 -0.175–0.188	-0.051	0.156	-0.019 -0.359–0.257	0.031	0.198	0.011 -0.361–0.421	**0.126**	**0.046**	0.143* **0.054–0.254**
Budget of the SSPP	0.061	0.041	0.056 -0.021–0.142	-0.005	0.088	-0.004 -0.178–0.169	-0.006	0.091	-0.005 -0.185–0.173	**0.115**	**0.062**	0.121* **0.007–0.237**
School’s interest in the SSPP	**0.715**	**0.036**	0.088* **0.034–0.143**	0.018	0.061	0.024 -0.100–0.136	0.063	0.070	0.077 -0.075–0.201	**0.091**	**0.049**	0.117* **0.012–0.188**
General teachers’ interest in the SSPP	0.053	0.032	0.068 -0.010–0.117	0.081	0.628	0.101 -0.042–0.205	0.072	0.078	0.090 -0.080–0.225	0.061	0.052	0.073 -0.051–0.170
Number of teachers actively participating in the SSPP	0.018	0.026	0.023 -0.033–0.069	0.024	0.041	0.032 -0.055–0.103	0.010	0.054	0.013 -0.094–0.115	-0.034	0.048	-0.042 -0.129–0.060
**Teacher perceptions factors**												
School smoking policy assistance	**0.094**	**0.028**	0.107*** **0.040–0.149**	**0.101**	**0.044**	0.122* **0.013–0.186**	0.066	0.057	0.072 -0.047–0.179	0.068	0.053	0.071 -0.037–0.172
Burden of preparing educational materials for the SSPP	**0.104**	**0.025**	0.114*** **0.053–0.155**	**0.071**	**0.046**	0.073* **0.020–0.161**	0.096	0.051	0.109 -0.005–0.198	**0.135**	**0.041**	0.152** **0.054–0.215**
Necessity of smoking prevention and cessation education	**0.225**	**0.030**	0.225*** **0.165–0.286**	**0.282**	**0.050**	0.292*** **0.182–0.382**	**0.221**	**0.059**	0.231*** **0.104–0.336**	**0.174**	**0.054**	0.162** **0.068–0.280**
Effectiveness of the SSPP	**0.259**	**0.045**	0.244*** **0.170–0.348**	**0.261**	**0.079**	0.224** **0.105–0.416**	**0.209**	**0.080**	0.201** **0.051–0.367**	**0.352**	**0.085**	0.347*** **0.184–0.520**
Smoking prevention effect on nonsmoking students	0.031	0.041	0.030 -0.049–0.113	-0.054	0.071	-0.051 -0.192–0.084	0.028	0.079	0.026 -0.126–0.183	**0.119**	**0.071**	0.112* **0.018–0.257**
Smoking cessation effect on smoking students	**0.134**	**0.031**	0.161*** **0.078–0.202**	**0.191**	**0.049**	0.231*** **0.095–0.285**	**0.181**	**0.067**	0.205** **0.050–0.312**	**0.186**	**0.058**	0.216*** **0.081–0.347**
R^2^	0.529					0.615			0.481			0.573		
Adjusted R^2^	0.517					0.584			0.438			0.543		
F	45.73***					19.75***			11.29***			18.86***		

SSPP: school-based smoking prevention program. SE: standard error.

* p<0.05,

** p<0.01,

*** p<0.001.

Considering all teachers participating in the SSPP, overall satisfaction was influenced by seven variables across two factors. Among these variables, the variable that most strongly influenced satisfaction was the effectiveness of the SSPP (β=0.244; 95% CI: 0.170–0.348), indicating that as the perceived effectiveness of the program increased, satisfaction also significantly increased.

For elementary school teachers in the SSPP, overall satisfaction was influenced by seven variables across three factors. Regarding personal factors, the beta coefficient for age was 0.141 (95% CI: 0.032–0.218), suggesting that higher age groups are associated with higher overall satisfaction with SSPP operation. Regarding the school characteristics factors, the beta coefficient for school size was -0.128 (95% CI: -0.186 – -0.024), implying that as school size increases, satisfaction with the SSPP operation decreases. The beta coefficient for the necessity of smoking prevention and cessation education was 0.292 (95% CI: 0.182–0.382), indicating the strongest influence.

For middle school teachers, one factor was significantly influenced by three variables. The beta coefficient for the necessity of smoking prevention and cessation education was 0.231 (95% CI: 0.104–0.336), indicating that teachers who perceived the need for education reported greater satisfaction compared with teachers who did not perceive such a need.

High school teachers’ satisfaction with the SSPP was significantly influenced by nine variables across two factors. Among the school characteristics factors, the beta coefficient for the program type was 0.143 (95% CI: 0.054–0.254). Regarding the teacher perceptions factors, the beta coefficient for the effectiveness of the SSPP was 0.347 (95% CI: 0.184–0.520), suggesting that it had the strongest influence on overall satisfaction among all variables.

## DISCUSSION

This study identified variations in the assistance provided by school smoking policies, the effectiveness of the SSPP, the smoking prevention effect on non-smoking students, the smoking cessation effect on smoking students, and overall satisfaction with SSPP operations across different school levels. Additionally, factors influencing overall satisfaction with SSPP operations varied depending on the school level of the teachers.

Satisfaction with the operation of the SSPP was highest among high school teachers, followed by elementary school teachers, and then middle school teachers; notably, middle school teachers reported below-average satisfaction levels. These differences in satisfaction across school levels can be attributed to the unique characteristics inherent to each level. Specifically, the middle school years are characterized by increased time spent with peers rather than parents, the emergence of secondary sexual characteristics; therefore, this a period that involves increased independence and sensitivity^20^. The sensitivity of middle school students also impacts teachers, such that the aggression and intensity of student-to-teacher violence is greater compared with other grade levels^21^. Thus, compared with elementary school teachers, middle school teachers perceive less effectiveness in education, resulting in lower job satisfaction, increased emotional exhaustion, and a higher depersonalization rate, ultimately leading to burnout^22^. Additionally, the present study showed that middle school teachers perceived less effectiveness in all aspects of the SSPP compared with elementary school teachers in terms of teacher perceptions factors, suggesting decreased overall satisfaction with program operations and potential teacher burnout.

Variables significantly influencing satisfaction across all school levels were the necessity of smoking prevention and cessation education, the effectiveness of the SSPP, and the smoking cessation effect on smoking students. Research indicates that positive educational outcomes are achieved when there is a perceived need and demand for education, as well as when teachers’ rates of satisfaction with education, autonomy, and educational development abilities are enhanced^23,24^. Teacher effectiveness, satisfaction with education, and quality of education are interrelated^25^; a decrease in one of these components can lead to decreases in the others, highlighting the importance of teacher effectiveness ^25^. The SSPP, driven by teachers who perceive the necessity of smoking prevention and cessation education, aims to achieve educational effectiveness^10^. This study found that the necessity of smoking prevention and cessation education had the greatest positive impact on satisfaction among elementary and middle school teachers. This finding aligns with research showing that elementary school teachers’ achievement of set goals, increases their life satisfaction and their satisfaction with educational values^26^. Similarly, the SSPP is predicted to be the result of teachers’ goal to protect elementary school students from harmful factors such as smoking^15,16,26^. For middle school teachers, an increased need for student education is correlated with higher satisfaction with educational content and a greater passion for teaching^27^. Conversely, for high school teachers, the perceived effectiveness of the SSPP had the greatest impact on overall satisfaction with SSPP operations. Considering that high school students’ smoking behavior resembles that of adults^28^, and the current smoking and e-cigarette use rates among high school students in South Korea are significantly higher (10.4% and 7.0%, respectively) than those rates among middle school students (2.3% and 2.0%, respectively), high school teachers face considerable pressure in implementing the SSPP. The achievement of educational goals significantly improves job satisfaction for high school teachers^29^. As such, high school teachers, who implement the SSPP under significant pressure to reduce high current smoking rates among students^10,28,29^, are likely to find that higher effectiveness ratings of the SSPP are correlated with increased satisfaction with the program’s operation.

The variables that solely affected the satisfaction of elementary school teachers were the teacher’s age, school size, and the assistance of the school’s smoking ban policy. Study results regarding job satisfaction factors for elementary school teachers indicate that as teachers age, their satisfaction tends to increase; seniority allows them to act as a bridge between novice teachers and students to achieve educational goals^26,30^. Furthermore, elementary students fear being reprimanded by teachers for not following rules, and strong district policies can increase students’ learning performance by almost two-fold^30,31^. Therefore, strong smoking bans in elementary schools could strongly deter student smoking initiation, thereby enhancing teachers’ satisfaction with SSPP operations.

Conversely, no variables exclusively influenced the satisfaction of middle school teachers regarding the SSPP operation. In this study, middle school teachers’ overall satisfaction was below average and the lowest among the three school levels. Middle school teachers in the United States had lower educational satisfaction compared with their elementary and high school counterparts^32^; middle school teachers in China also exhibit the highest levels of dissatisfaction^33^. The middle school period, is characterized by increased sensitivity, presents challenges for teachers in understanding and guiding students’ behavioral patterns because their learning styles may not be actively accommodated^22,32^. Teachers may struggle to recognize the differentiated educational values and characteristics of middle school students^21^, leading to the conclusion that no specific variables affect middle school teachers’ satisfaction. This underscores the need for further exploration and research focused on middle school students from an educational perspective to enhance middle school teachers’ satisfaction with SSPP operations.

For high school teachers, variables exclusively influencing their satisfaction with SSPP operations included school location, SSPP type, SSPP budget, the school’s interest in the SSPP, and the smoking prevention effect on non-smoking students. Smoking rates among youth are 4.7% in the capital region, 5.4% in metropolitan cities, and 6.3% in local government areas, demonstrating an increasing trend in smoking rates toward rural regions^9^. Thus, localities with higher smoking rates constitute a significant source of dissatisfaction for teachers attempting to mitigate smoking rates. High schools, facing higher smoking rates, receive more budgetary resources^9^; this is based on research suggesting that high budget allocations for educational programs enhance efficacy^34^ and satisfaction among high school teachers. It is also speculated that advanced-type schools, which receive more budgetary resources than basic-type schools^9^, positively impact teachers’ satisfaction with SSPP operations. Therefore, it is critical for schools to actively support high schools by allocating more budgetary resources for the SSPP in future allocation cycles and to actively proceed with SSPP implementation.

### Limitations

First, because this study analyzed only the 2022 survey results on the operation of the SSPP, it is difficult to generalize the findings to all teachers. Furthermore, since there is no globally standardized smoking cessation education program, the results of this study cannot be generalized to SSPP participating teachers in other countries. Second, the use of self-administered survey data may have introduced recall bias, potentially influencing the results. Third, this study was based on secondary data, which limits the analysis of additional teacher characteristics that may affect SSPP operational satisfaction, such as self-assessment of teaching abilities^12-14^ and perceptions of novel forms of tobacco^35^. Similarly, we did not examine teacher smoking characteristics that could influence student smoking behavior, such as teacher smoking habits or students witnessing teachers smoking, which could potentially encourage student smoking behavior^36^. Future studies should analyze more factors that may affect SSPP operations, including teacher smoking behavior and recent forms of smoking, such as e-cigarette use patterns, to adjust for the increasing diversity of smoking behaviors.

## CONCLUSIONS

This study categorized teachers participating in the SSPP according to school level to identify factors that commonly and exclusively influence their satisfaction with program operations. Across all teachers, the effectiveness of the SSPP emerged as the factor most strongly influencing satisfaction. For elementary and middle school teachers, the necessity of smoking prevention and cessation education was the factor most strongly influencing satisfaction. Conversely, for high school teachers, the effectiveness of the SSPP was the factor most strongly influencing satisfaction. The variation in SSPP satisfaction according to school level was attributed to differences in student temperament at each level, which led to variations in the perceived necessity of the program and its effectiveness. Therefore, the establishment of a healthy environment for the SSPP through meticulous observation, targeting both teachers and students, and implementation of specialized strategies tailored to each school level, are imperative.

## Data Availability

The data supporting this research are available from the Korea Health Promotion Institute on reasonable request. The data are not publicly available to protect the confidentiality of the research participants.
